# Working with migrants and refugees - Ethical principles and dilemmas in psychiatry

**DOI:** 10.1192/j.eurpsy.2023.88

**Published:** 2023-07-19

**Authors:** M. Schouler-Ocak

**Affiliations:** Psychiatrische Universitätsklinik der Charité im St. Hedwig-Krankenhaus, Berlin, Germany

## Abstract

**Abstract:**

More than 100 million people are forcibly displaced including refugees, internally displaced persons and asylum seekers who have fled their homes to escape violence, conflict, and persecution. The vulnerable group of refugees and forcibly displaced people have a high burden of mental disorders, including post-traumatic stress disorder (PTSD), depression, idioms of distress, and prolonged-grief disorder, which are highly related to the load of traumatic circumstances surrounding refugees associated with human rights abuses, lack of human needs, and separation from others and many refugees are severely traumatized and suffer a variety of symptoms, and not all seek help. Their mental health problems are of emergency nature place a huge burden upon services which are difficult to deliver. This implies that mental health professionals and patients are more likely than ever to come from different cultural backgrounds. To have access to mental health care is often a challenge for them and most of them do not seek help. Unfortunately, these vulnerable groups are not treated equally according to ethical principles of mental health professionals. This presentation will focus on these dilemmas and discuss them.

**Disclosure of Interest:**

None Declared

